# Supplementary material for: Structural and Functional Impairments of Reconstituted High-Density Lipoprotein by Incorporation of Recombinant β-Amyloid42
Source: Molecules. 2021 Jul 16;26(14):4317. doi: 10.3390/molecules26144317 (PMC8303321; doi:10.3390/molecules26144317)
Supplement: Supplementary file 1 [file molecules-26-04317-s001.zip › molecules-1276323-supplementary.pdf]

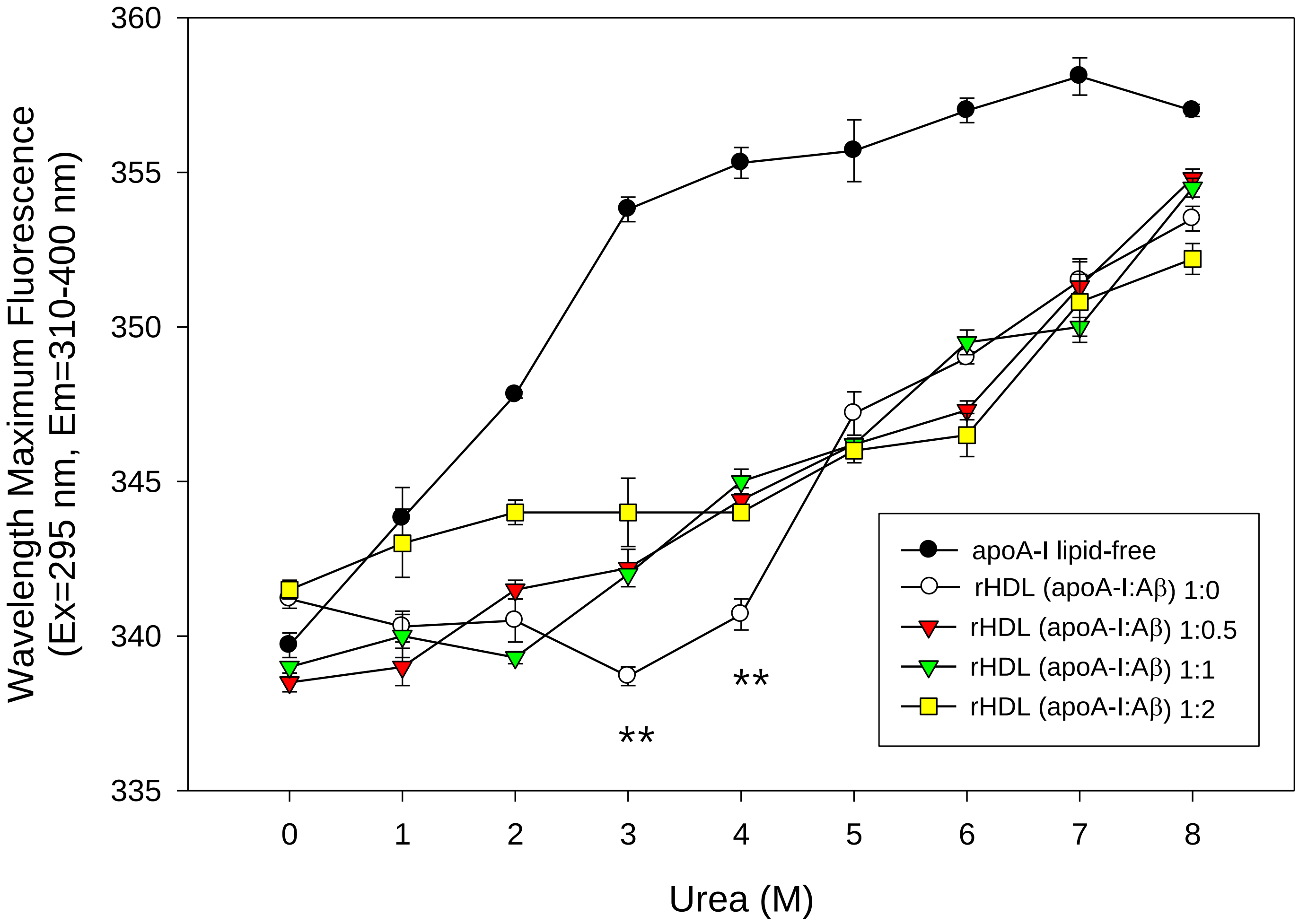

Suppl. Figure S1. Isothermal denaturation of apoA-I-rHDL containing Aβ. Exposure of Trp fluorescence was detected by fluorospectroscopy (Ex=295 nm, Em=310-400 nm). **, $p<0.01$ between rHDL (apoA-I:Aβ, 1;0) and rHDL(apoA-I:Aβ, 1;2) under presence of 3 M and 4 M urea
